# Innovations in Smart Packaging Concepts for Food: An Extensive Review

**DOI:** 10.3390/foods9111628

**Published:** 2020-11-07

**Authors:** Emanuela Drago, Roberta Campardelli, Margherita Pettinato, Patrizia Perego

**Affiliations:** Department of Civil, Chemical and Environmental Engineering (DICCA), Polytechnique School, University of Genoa, Via Opera Pia 15, 16145 Genova, Italy; emanuela.drago@edu.unige.it (E.D.); roberta.campardelli@unige.it (R.C.); p.perego@unige.it (P.P.)

**Keywords:** food safety, food product shelf-life, packaging technologies, polymers packaging, additives, sensors, nanocomposites

## Abstract

Innovation in food packaging is mainly represented by the development of active and intelligent packing technologies, which offer to deliver safer and high-quality food products. Active packaging refers to the incorporation of active component into the package with the aim of maintaining or extending the product quality and shelf-life. The intelligent systems are able to monitor the condition of packaged food in order to provide information about the quality of the product during transportation and storage. These packaging technologies can also work synergistically to yield a multipurpose food packaging system. This review is a critical and up-dated analysis of the results reported in the literature about this fascinating and growing field of research. Several aspects are considered and organized going from the definitions and the regulations, to the specific functions and the technological aspects regarding the manufacturing technologies, in order to have a complete overlook on the overall topic.

## 1. Introduction

Traditional food packages are passive barriers designed to delay the adverse effects of the environment on the food product [1]. Modern food packages, instead, besides the passive role of containment, protection, and marketing of the product are characterized by an active function that allows packages to play a dynamic role in food preservation (during processing and storage), retaining the safety and quality of food throughout the distribution chain [2]. In other terms, the key safety objective for traditional packaging materials coming in contact with food is to be as inert as possible; whereas the innovative packaging systems concepts are based on the useful interaction between packaging and the environment inside, to provide active protection to the food [3]. Many terms are used to describe innovative packaging technologies such as “active”, “interactive”, “smart”, “clever”, “intelligent”, “indicators”, etc. [4]. These terms often lack in a clear definition and are interchangeable in some literature. Therefore, it is important to differentiate their meanings [5]. Intelligent and active packaging are two very different concepts united by the fact of having started a new way of conceiving food packaging. The main difference is that intelligent packaging does not act directly on food with any action other than monitoring the condition of the packaged product, while active packaging acts on the environment surrounding food to increase the shelf-life. Therefore, active packaging is the component that takes some action, while intelligent packaging is the component that senses and shares the information [6]. Intelligent and active packaging can, almost inevitably, work in synergy to create what is called a “smart” packaging [7].

Considering the manufacturing techniques for innovative packaging production, it has to be considered that most of the time the package is produced in the conventional way such as extrusion, injection molding, injection stretch blow molding, casting, blown film, thermoforming, foaming, blending, and compounding. The active component can be directly incorporated in the polymer-based package matrix and/or film otherwise sachets and pads can be inserted in the package. The intelligent component can be instead integrated in the primary or secondary packaging. However, also polymer packaging manufacturing innovations are interesting, in order to follow the progress emerging in packaging composition and structure.

From a market point of view, the position covered by active and intelligent packaging in Europe is far behind foreign markets, in particular Japan, USA and Australia, where these products are widely commercialized [8]. This lag was often attributed to inadequate and very strict European regulation of food packaging [9]. To date, the only regulation entirely dedicated to intelligent and active materials for food packaging is Regulation 450/2009 [10], which establishes specific requirements on the use and authorization of active and intelligent materials as materials intended to come into contact with foods.

**Figure 1:**
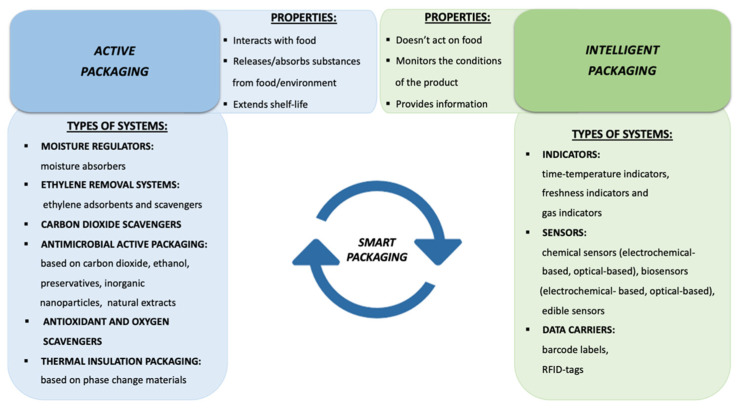
Active and Intelligent packaging classification and their main properties.

The aim of this review is to present an overview of recent innovations in food packaging with the objective of providing clear definitions and classifications of each kind of active and intelligent packaging, and related manufacturing technologies up to commercial applications. A comprehensive diagram of all the categories of active and intelligent packaging detailed in this work is shown in Figure 1, from which it is also possible to deduce the different function that these two types of packaging perform with respect to packaged foods.

## 2. Definition and Regulatory Aspects

In Europe, active and intelligent packaging must be in agreement with the Legislation about food-contact materials, which includes the evaluation of the overall migration limits (OMLs), specific migration limits (SMLs) and toxicological properties. In particular, the Regulation 1935/2004/EC [11] establishes in art. 3 that any material or article intended to come into contact directly or indirectly with food shall be manufactured in compliance with good manufacturing practice, in such a way that under the normal and foreseeable conditions, their constituents do not migrate toward food in quantities that could represent a risk of human health that could change food composition or that could deteriorate food organoleptic properties. National and International regulations, specific for categories of materials are available, among them Regulations (EU No. 10/2011 [12] and Regulation (EU) 2016/1416 [13], amending and correcting the former, are of particular interest in the field of packaging. In addition, the Regulation 1935/2004/EC in article 4 regulates the labeling which must indicate that the parts are non-edible and that intelligent materials shall not give information about the condition of the food which could mislead consumers [11]. The Commission Regulation 450/2009/EC [10] specifically deals with active and intelligent materials and articles intended to come into contact with food. In particular, it is emphasized that intelligent packaging systems must not release their components into food. The intelligent component is allowed to be placed on the external side of the package and the contact with food may be avoided by a functional barrier that prevents the migration of substances. The functional barrier allows the use of unauthorized substances, under the respect of defined criteria and of the established migration limits. Furthermore, it is also specified that the risk related to the employment of technologies such as nanoparticles should be analyzed for the specific application until more information is available regarding their use and their possible interactions with food and with human health [10]. Concerning active packaging, art. 5 indicates that components which may be added to food packaging with active function must be listed in the Community list of authorized substances. On each of the listed substances, the European Food Safety Authority (EFSA) performed a risk and safety assessment including the migration of the active agents, the migration of their reaction products and the evaluation of their toxicological properties. In general, active packaging can be classified into non-migratory and migratory. The former implies that the action of active agent is carried out without intentional migration of non-volatile or volatile compounds from packaging into food. Nevertheless, the active agent in the packaging may deliberately be intended to be released into food. In this case, the overall migration limit of the packaging may be exceeded, so the Regulation implies two exceptions. Indeed, if the active function is not a specific feature of the passive material, the amount of released active agent should not be included in the evaluation of overall migration. In addition, the migration of the released active substances can exceed the specific migration limit if its concentration in food is in compliance with the applicable food law. Furthermore, the active substance intentionally released in food, or that have a technical effect, must comply with the Regulation 1333/2008/EC [14], concerning direct food additives. For already approved substances which are incorporated in active materials by means of techniques such as grafting and immobilization, the manufacturer is expected to perform a safety evaluation and to verify the stability of the substances when chemical reactions, degradation or decomposition are expected to occur. Furthermore, the active substances should not mislead the consumer (e.g., masking spoiled food), and they must be indicated in the label associated with the sentence “do not eat” or a symbol to prevent the nonedible part to be erroneously ingested. An extensive overview about regulation aspects of food active packaging in Europe is reported by Restuccia et al. (2010) [15].

Considering different jurisdictions, a different approach compared to the European one can be observed. The introduction of active and intelligent packaging into the Japanese market is already consolidated, but specific regulations related to these two categories of packaging are still missing. Indeed, safety of active and intelligent packaging is ensured by the application of the Japanese Food Sanitation Law of 1947, together with the Food Safety Base Law of 2003. Generally, to introduce active and intelligent packaging materials to the Japanese market, risk assessment criteria are adopted including also compliance with foreign regulations, such as the European Union or the United States [16]. For US regulatory concepts about food-contact materials, toxicological justification is greatly minimized by exposure assessments compared to European legislation. Active and intelligent packagings are considered almost like conventional packaging. Food-contact materials are subjected to regulatory clearance before placing on the market by the United States Food and Drug Administration if they are considered “food additives” under the Federal Food, Drug, and Cosmetic Act, while as long as the material in the active or intelligent packaging is intended neither to add any substance to the food, nor to have a technical effect in the food (so-called “indirect additives”), there are no specific regulations for these components, which are simply regulated like all other food-contact substances [15]. The Canadian food packaging regulatory system is based on high safety standards and is similar to the one of US. The crucial difference between the two systems concerns migrating food packaging components, which are not considered to be food additives, legally requiring Health Products and Food Branch (HPFB)preclearance [17].

## 3. Active Packaging

Active food packaging represents an improvement in the function ascribed to the classical packaging, aiming to increase product shelf-life by interacting with food or the environment surrounding food. A thorough definition of “active materials and articles” is given by European Regulation 450/2009/EC [10]. It defines them as “designed to deliberately incorporate components that would release or absorb substances into or from the packaged food or the environment surrounding the food” [10]. Indeed, conventional food packages are usually intended as passive barriers, which protect food by segregation against environmental contaminations, while active packaging promotes an active action of the packing material providing enhanced food conservation. This active action may be due to particular features that belong to the packaging material or to active agents, which are added to the passive barrier in order to work as absorbers, emitters or releasers of compounds that are able to play a pivotal role in food preservation. Thus, the main target of active packaging is to prevent microbial and chemical contamination, as well as to maintain visual and organoleptic properties of food.

Table 1 shows some examples of commercially available active food packaging for each of the categories which will be discussed in the following paragraphs.

### 3.1. Moisture Regulators

Moisture absorbers are active non-migratory packaging, working as controllers of excess moisture. High content of water into the package may be provided by several causes, such as trapping during packaging process, release due to temperature fluctuations, respiration of fresh products, low vapor permeability of the package, etc. Excess water reduces food shelf-life, since promotes microbial proliferation inducing undesired changes in food quality, particularly in dry food. In addition, presence of liquid inside the package of raw fish and meat, due to dripping or formation of foggy film in packaging of fresh fruit and vegetables, results into a low appeal for customers [18,19]. The main purpose of moisture absorbers is the reduction of food water activity by using hygroscopic substrates or substances, providing an environment less suitable for mold, yeast and bacteria growth and spoilage. Some packaging materials used as passive system are inherently moisture scavengers due to their hygroscopicity. According to the Regulation 450/2009/EC [10], these kind of materials and articles, which work on the basis of the natural constituents only and are not designed to deliberately absorb substances, cannot be defined as active systems (e.g., pads composed of 100% cellulose). Nevertheless, if they contain components designed for the scope, they fall under the definition of active packaging. Moisture scavengers can be defined as “relative humidity controllers” if they provide a reduction of humidity in the headspace, while “moisture removers” absorb liquids exuded from food and are usually placed on the bottom of fresh products and meat packages [20]. Examples of moisture absorber commercially available are absorbent pads, sheets, trays and blankets [18,20,21,22], as summarized in Table 2. The structure comprehends two layers of microporous non-woven plastic film in polyethylene, polypropylene, etc. containing the active substance, such as superabsorbent polymer, silica gel-based adsorbents, sodium chloride, starch copolymers, etc. [21,22]. For package design, it is important that the active agent maintains its properties after the package processing and must not interfere with plastic properties.

In addition, the polymer containing the desiccant has to be water-permeable [23]. Nevertheless, for high water content products, moisture absorber should be carefully designed in order to avoid undesired food dehydration [24]. Sachets containing desiccants as calcium oxide, calcium chloride, molecular sieves, natural clays, and silica gel are usually employed for humidity control in low-level moisture food, for instance in dry food packages [25]. In addition, moisture absorbing material can be dispersed into the polymeric matrix or between two layers of plastic films constituting the package [18]. Inorganic compounds used as desiccants include materials such as silica gel, bentonite, calcium sulfate, molecular sieves, which are able to retain increasing amounts of water as the humidity increase, or metal oxides, such as calcium, barium, and magnesium oxides, which irreversibly react with water providing the corresponding oxides [23]. Organic-based absorbers are also reported such as fructose [26], sorbitol and cellulose and modified starch [25]. Liu et al. [27] reported the application of graphene oxide papers as desiccant for food preservation. They studied the moisture adsorption capacity of graphene oxide papers with different conditions of surface functionalization, showing promising results for applications related to food preservation. An extensive review about moisture absorbers was provided by Gaikwad et al. [18].

### 3.2. Ethylene Removal Systems

Ethylene is a pure unsaturated hydrocarbon, odorless and colorless. It is a hormone naturally produced by plants and plays an important role in their growth cycle, respiration rate, somatic embryogenesis, seed germination, root growth and development. On the one hand, its action can lead to an appropriate ripening process that prepares fresh produce to the market (e.g., de-greening of lemons), on the other hand the acceleration of ripening and degradation of chlorophyll can cause a deterioration of the quality and reduce the shelf-life during postharvest storage of fresh products [20,28]. Climacteric fruit and vegetables produce high amount of ethylene, aldehydes, and other gases during ripening, which further promote the ripening process. The reduction of ethylene in the package atmosphere by active systems can slow undesired effects on these products [29]. The presence of just 1 ppm of ethylene in the package is sufficient to trigger climacteric fruit ripening [30]. Conversely, the application of ethylene removal systems is not common in packaging of non-climacteric fruit (cherry, strawberry, pineapple, etc.), since they do not require ethylene gas during ripening [28]. Ethylene removal can be carried out by ethylene absorbers, which physically absorb and hold its molecules, and scavengers, acting by chemical reactions.

#### 3.2.1. Ethylene Adsorbents

Ethylene adsorbents include silica, zeolite, montmorillonite, cloisite, activated carbon and Japanese Oya clay [22,31,32]. In general, commercial ethylene adsorbents can be provided in sachets or could be incorporated into a plastic film structure, commonly used in fresh produce packaging [20,33]. Recently, Gaikwad et al. [34] studied alkali-treated halloysite nanotubes as ethylene absorber compared to raw halloysite nanotubes. The alkaline treatment provided an increase of the pore size of natural halloysite allowing a larger and faster adsorption capacity of ethylene than raw halloysite nanotubes. A novel work [35] provided useful data for the design of new adsorbent based on the interesting properties of Yttrium doped graphene oxide. The adsorption properties of H_2_O, CO, and C_2_H_4_ molecules on the surface of the graphene oxide and Yttrium doped graphene oxide were investigated by the density functional theory calculations. Results showed as the substituted Yttrium atom can affect the electronic properties of graphene oxide molecule, leading to a noticeable increase in the adsorption energies.

#### 3.2.2. Ethylene Scavenger

The most widely used ethylene scavengers is made by potassium permanganate (4–6%) [32] supported on inert matrices, such as alumina or silica gel [33]. This compound is not integrated in surfaces in contact with food because of its toxicity [29], so it is embedded in minerals or nanoparticles to enhance its scavenging ability and it is usually enclosed in permeable sachets [28]. Moreover, KMnO_4_-based scavengers are also available in the forms of tube filters, blankets, and films [32]. Mechanism of action involves the conversion of ethylene gas by oxidation into ethylene glycol, carbon dioxide and water [22]. Due to their high surface activity and reactivity, nanoparticles can be used to oxidize ethylene into water and carbon dioxide by photocatalytic reaction, using silver, titanium dioxide, zinc oxide, copper, and palladium [28]. Chitosan-titanium dioxide nanocomposite film was developed by Siripatrawan and Kaewklin [36], who aimed to reduce the problems related to the spontaneous agglomeration observed for TiO_2_ nanoparticles, and to produce an active packaging with both ethylene scavenger and antimicrobial properties. Chitosan film containing 1% TiO_2_ showed suitable water barrier and ethylene degradation properties, as well as antimicrobial activity. Supplementary details about ethylene scavengers can be found elsewhere [28,32].

### 3.3. Carbon Dioxide Scavengers

Presence of carbon dioxide into food packages is generally beneficial and this aspect will be discussed in following paragraph. Nevertheless, an excess of carbon dioxide can induce undesirable effects on food and packaging. Excess of carbon dioxide could be due to the metabolism of the product and of microbial contaminant, being CO_2_ one of the main results of catabolic reactions that occur in biological systems. For instance, packaged non-pasteurized or non-sterilized fermented foods, such as kimchi, yogurt, cheese, and soy paste [37], continue the microbial activity during storage and distribution. The microbial activity produces high levels of CO_2_ inside the package, leading to its potential collapse or to undesirable changes in texture and flavor of food, such as discoloration, off-flavor development, and tissue breakdown. Examples of foods, whose quality can be affected by excess of carbon dioxide are potato, lettuce, onion, cucumber, cauliflower, artichoke, apricot, peach, apple, and carrot [38]. Also roasted coffee is able to release high amount of carbon dioxide, which may cause the package to burst. Due to the low permeability of some packaging to CO_2_ and to overcome its accumulation, scavengers placed into the food package in form of sachets were developed. Calcium oxide and hydrating agents such as silica gel enclosed in porous sachets allow the reaction between water and calcium oxide forming calcium hydroxide, which finally reacts with CO_2_ producing calcium carbonate [29]. Other absorbers consist of sodium hydroxide, potassium hydroxide [21] in form of sachets or granules or physical absorbers (zeolites, activated carbon) in form of beads and powder [38]. Commercially available solutions and applications of carbon dioxide scavengers are reported by Han et al. and by Verneiren et al. [38,39] and extensively treated by Lee [37].

### 3.4. Antimicrobial Active Packaging

Food shelf-life is strongly affected by the presence of microorganisms. Contamination could occur at every stage of the supply chain, whenever food is exposed to the environment and its outcomes can range from simple alterations of sensory features to serious health hazards for consumers. Among the technologies developed to hinder this issue, antimicrobial packaging can be an effective tool to improve food safety, extend food shelf-life and reduce food waste and economic losses. Active emitters contained in packaging aim to provide a controlled release of compounds, able to ensure a right level of humidity, inhibition of harmful microorganisms and prevent bacteria spoilage [40].

#### 3.4.1. Carbon Dioxide

Carbon dioxide has a well-known antimicrobial effect on several microorganisms and it is used in modified atmosphere packaging to prolong food shelf-life. Nevertheless, since CO_2_ permeability through some packaging materials is higher than oxygen and it can also dissolve in food, carbon dioxide generators are needed to maintain the CO_2_ concentration to a desired value and to avoid packaging deformation [38]. The antimicrobial activity of carbon dioxide is strictly related to its rate of solubility and the amount dissolved in food, which increases as temperature decreases, and in food packaging it is proportional to the gas partial pressure in the headspace [20]. For fresh products partial pressures of CO_2_ higher than 0.1 atm are able to inhibit the respiration rate and microbial growth on food surface [23]. The formation of carbonic acid within the cell, decreasing the intracellular pH and activities, inhibition of decarboxylating enzymes, non-specific inhibition of susceptible non-decarboxylating enzymes, inhibition of membrane functions are all possible mechanisms of CO_2_ antimicrobial activity [41]. Sachets or pads with double action of CO_2_ emitters/O_2_ scavengers are usually employed. Iron carbonate (II) and metal halide as a catalyst are conventionally used [40]. Ferrous carbonate, combinations of sodium bicarbonate and citric acids, or ascorbic acid and sodium bicarbonate are commercially solutions to obtain CO_2_-releasing system [20,38].

#### 3.4.2. Ethanol

Ethanol is an antimicrobial agent able to inhibit yeast and bacteria growth. It is particularly effective on molds, and its effect in prolonging shelf-life of bakery products by direct spraying was widely demonstrated. Emitting sachets or films containing food grade ethanol provide the exchange of ethanol with water vapor in the package headspace. To mask the odor of ethanol, sometimes flavors are added to the sachets. Examples of commercial ethanol generators are listed in Table 1, while further examples were provided by Vermeiren et al., Suppakul et al., and Sung et al. [39,42,43]. The rate of ethanol release depends on carrier water permeability, amount of ethanol into the sachet, food water activity and ethanol permeability through film material. In the latter case, ethanol imbedded films usually require additional layers to provide a sustained release. Recently, Mu et al. [44] proposed an ethanol gel, obtained by gelation reaction between ethanol and sodium stearate, adsorbed on diatomite, in order to enhance the release of ethanol emitter. The main disadvantage of ethanol emitters is its absorption on food, which can be reduced to insignificant values by heating or microwaving the product. Food products consumed without being heated may contain residual ethanol and might cause regulatory problems [25,45].

#### 3.4.3. Preservatives

Chlorine dioxide and sulfur dioxide enclosed in sachets and pads attached to the internal part of the package are volatile agent with antimicrobial action. Commercial products are reported by Ozdemir et al. and Sung et al. [25,43]. Non-volatile preservatives with antimicrobial activity include weak acids and their salts such as acetate, sorbate, benzoate, and propionate, which probably exert the antimicrobial activity being transported in the undissociated form into the plasma membrane, where the higher pH provide the dissociations in ions that cannot return through the plasma membrane. The antifungal activity of potassium sorbate in films was reported in several studies [46].

#### 3.4.4. Inorganic Nanoparticles

Moreover, inorganic nanoparticles composed of metal ions of silver, copper, gold, platinum, selenium [47] and metal oxides such as TiO_2_, ZnO, MgO, and CuO are used for the production of active packaging, obtained by the incorporation of such materials into adsorbent pads or plastic films. Even if the mechanism of action is still under debate, the antimicrobial activity seems to be due mainly to the release of metal ions and the formation of reactive oxygen species, which provoke deadly damages to the wall and membranes of microbial cells [46,48]. The small size of nanoparticles implies a high surface-to-volume ratio, resulting in an enhance of metal reactivity as photocatalysts and improving the interactions between metal nanoparticles and microbial membranes [20]. Recently a review by Basavegowda et al. [49] explored the advantages of bimetallic and trimetallic nanoparticles compared to monometallic ones and their potential application in food packaging. They are hybrid nanostructured material showing improved thermal, mechanical and gas barrier properties and higher antimicrobial activity compared to monometallic nanoparticles [49]. However, the use of such inorganic nanoparticles should be carefully selected, basing on the type of food and the properties of packaging film (barrier properties, transparency) that can be affected. Furthermore, some concerns related to potential toxic effects on human health due to migration to food, dermal contact, and inhalation, are presently increasing [20,46], and depends on the chemical composition of nanoparticles, size, surface chemistry, solubility, and hydrophobicity [49].

### 3.5. Synthetic Antioxidants and Oxygen Scavengers

Food products are generally sensitive to oxygen, which induces undesired changes to the organoleptic properties, such as color modifications, development of off-flavors, as well as deterioration of nutritional properties, and supports microbial growth [33,50]. Consequently, great importance is given to strategies aiming to minimize oxygen content in the headspace of food packages. Vacuum and modified atmosphere packaging are two methods that proved to be effective in prolonging shelf-life of some foods. Nevertheless, both of them cannot impede the permeation of oxygen over time from the external environment and are also able to reduce the amount of oxygen in the package up to only 0.5–2 vol.%, while lower values can be achieved by oxygen scavengers [50,51]. The control of oxygen levels is obtained using synthetic antioxidants and oxygen scavengers.

Oxygen scavengers are used in packaged food products in sachets, bottle crowns, labels, plastic films, and trays [50]. One of the most used solutions available in the market employs metallic scavengers, which carry out oxygen removal by chemical reactions. For instance, in iron-based scavengers (iron powder, activated iron, ferrous oxide, iron salt) the reduced metal oxidation occurs in presence of moisture or Lewis acids [51]. The rapid rate of oxidation of iron, can be further increased by using nano-iron particles blended with activated carbon, sodium chloride and calcium chloride [20]. However, some disadvantages of iron-based scavenging systems are related to potential contamination of food due to accidental breakage, to interferences with inline metal detectors and to the inhibition of heating by microwave ovens [52]. Other metals employed are platinum and palladium as efficient catalysts for the conversion of hydrogen in water. For this purpose, the atmosphere needs to be modified in order to hold high pressures of molecular hydrogen and the metal catalyst is used to improve the reaction also in presence of small amount of oxygen. Nevertheless, as reported by [53], due to the flammability of hydrogen, there is a maximum of oxygen that can be removed (2.5 vol. %) when hydrogen is introduced in the modified atmosphere. An alternative involves hydrogen-evolving compounds, such as calcium hydride or sodium borohydride, which allow a controlled hydrogen release, and which can be easily inserted in the cap of bottles, while more difficulties are met in their incorporation in films [54]. In addition, films based on oxidizable transition metals such as copper, zinc, magnesium, manganese, aluminum, or titanium, and nanocrystalline titanium particles are also reported [52]. Commercial solutions and mechanisms of action of the most common oxygen scavengers in food packaging as well as new approaches in this field can be found in the recent reviews published by Dey and Neogi [51] and Gaikward et al. [52].

The most common synthetic antioxidants comprise phenolic compounds as butylated hydroxytoluene, butylated hydroxyanisole and tert-butylhydroquinone and propyl gallate, which are hydrogen donating free radical scavengers [33]. They are widely used in food active packaging in order to prevent lipid oxidation, thus they are extensively applied to enhance the shelf-life of fat and fat containing products [21]. In addition, although they are generally used as antioxidants, they have shown also antimicrobial activity [42]. However, these compounds are suspected to have potentially toxic and carcinogenic effects, thus the research is more oriented toward the application of natural antioxidants [33], which will be discussed in this review in the following paragraphs.

### 3.6. Agents for Active Packaging from Natural Products

The use of naturally occurring agents for the fabrication of active food packaging is the trend to which the research is currently approaching. Natural antioxidant and antimicrobial agents are perceived as safer than synthetic ones by consumers, thus different natural compounds have been proposed for active packaging purposes and the interest of the scientific community in this field is continuously increasing.

#### 3.6.1. Bacteriocins and Enzymes

Bacteriocins, such as Pediocins, Nisins, Enterocins and Sakacins, are peptides produced by bacteria ribosomal synthesis, which gained strong attention by food industry due to their antimicrobial effect [55], even at low concentrations [56]. The main bacteriocin producers are lactic acid bacteria, so these naturally occurring compounds are present in several fermented food. They are able to inhibit many pathogenic microorganisms, but the mechanism of action is still not completely known. Bacteriocins produced by each strain are active only against their competing bacteria, since the originating bacterial cells possess specific immunity mechanism toward their bacteriocins [57]. Some hypotheses related to the mechanism of action were evaluated by recent studies and discussed in a recent review by Santos et al. [57]. Such mechanisms include the disturbance of bacterial metabolism, interaction with intracellular targets, inhibition of the synthesis of nucleic acids and proteins, interferences with the formation of cellular components, in which the cationic characters of the antimicrobial peptides play a crucial role in their adsorption on microbial cell surfaces.

Bacteriocins produced by lactic bacteria show properties that make them attractive for active packaging applications. Indeed, they are substances generally recognized as safe and are inactive and nontoxic on eukaryotic cells and on consumers. In addition, bacteriocins have little influence on consumer’s gut microbiota, being inactivated by digestive protease, while show broad antimicrobial activity against many food-borne pathogenic and spoilage bacteria. Furthermore, they do not provide any alteration of the organoleptic properties of food as they are taste-, odor-, and colorless [58]. Finally, they are active in wide ranges of pH and temperatures [59]. The last aspect plays an important role from an industrial point of view, since standard melting blending technological processes are not suitable for thermosensitive compounds [56]. Nisin is a commercially acceptable food grade bacteriocin, active against Gram-positive bacteria such as *Listeria monocytogenes*, *Clostridium botulinum*, *Staphylococcus aureus* and *Bacilli.* It is allowed for use in pasteurized cheese and liquid eggs and commercially used in a range of foods including dairy, eggs, vegetables, meat, fish, beverages, and cereal products [60]. Holcapkova et al. [56] found that the antimicrobial activity of nisin against *Micrococcus luteus* is temperature-resistant, indeed they showed that the polylactic acid (PLA)-based films displayed antimicrobial properties even when they were thermally treated above the melting temperature of PLA, but losing the 25% of their activity under treatment at 160 °C and more than 60% at 180 °C for 5 min. Pediocin was also reported to be a remarkable natural biopreservative to overcome the post-processing contamination of meat products against *L. monocytogenes* [61]. According to literature, nisin and other bacteriocins such as pediocin and lacticin are ineffective against molds, yeasts, and Gram-negative bacteria, with the only exception given by a few strains. Incorporating chelating agents such as Ethylenediaminetetraacetic acid (EDTA) was found to increase the effectiveness of bacteriocins in food packaging application [62]. However, in a recent work, the bacteriocins extracted from standard *Pediococcus acidilactici* culture and the isolate, *Enterococcus faecium*, showed antibacterial activity against both Gram-positive and Gram-negative bacteria [55]. In addition, Woraprayote et al. [59] reported that Bacteriocin 7293, a novel bacteriocin from *Weissella hellenica* BCC 7293, with polylactic acid/sawdust particle (PLA/SP) film provided both Gram-positive (*Listeria monocytogenes and Staphylococcus aureus*) and Gram-negative bacteria (*Pseudomonas aeruginosa*, *Aeromonas hydrophila*, *Escherichia coli and Salmonella Typhimurium*) inhibition in pangasius fish fillet. In addition, bacteriocins demonstrated additive or synergistic effects when used in a combination with other bacteriocins or with other preservatives or phenolic compounds [63].

Enzymes are also currently applied as antimicrobial agents in food packaging. In particular, lysozyme is a naturally occurring enzyme with activity against cellular structure specific to bacteria, since it is able to damage the structural integrity of the cell wall, resulting in the lysis of bacterial cells [60]. This enzyme exhibits antimicrobial activity against Gram-positive bacteria but not on Gram-negative bacteria, but susceptibility of Gram-negative bacteria could be enhanced by the addition of chelating agents such as EDTA [64]. Another enzyme that can be used as an active agent is Glucose oxidase, whose reaction products, yielding hydrogen peroxide from glucose and oxygen, show antimicrobial power [62]. However, the application of this enzyme in packaging is limited by the cost of enzyme and the requirement of glucose, whose amount in many foods in not sufficient [64]. Both bacteriocins and enzymes were employed as directly incorporated in polymer matrix, as coated onto material’s surface or by immobilization in active packaging. The first method is used with biodegradable polymers such as carbohydrates and proteins, when thermal processing is not used, due to the potential loss of antimicrobial activity. Alternatively, coating by immersing or contacting the film with a peptide solution or by solvent casting can be performed. Immobilization can be achieved by physical or chemical methods [57] and, due to the low tolerance of enzymes to high temperatures, immobilization is the option for the application of enzymes to antimicrobial packaging [64] and can improve the stability of bacteriocins [55,63].

#### 3.6.2. Phytochemicals

Among natural food preservatives, essential oils and other extracts from plants, herbs, and spices, deserve particular attention being able to exhibit great antimicrobial and antioxidant potential [65]. Essential oils are volatile substances because of the high content of low molecular weight aromatic compounds. They are biologically produced by plants as secondary metabolites and exert antimicrobial activity due to the high concentration of phenolic compounds (up to 85%), which provoke damages to bacterial enzyme systems, genetic material, and cell membrane phospholipid bilayers [48]. The main drawback of the use of essential oils and their components as antimicrobial agents is the high concentration required [33]. Most of essential oils extracted from plants and spices are classified as Generally Recognized As Safe but, due to their strong flavor potentially changing organoleptic features of food, their incorporation in films can be preferred to their direct use as food additive [66]. Examples of natural sources of essential oils proposed for active packaging applications are garlic [67,68], cinnamon [69], lemongrass [70], oregano [71], rosemary [72], thyme [73] and bergamot [74]. Antioxidant and antibacterial packaging materials with essential oils can be obtained by direct mixing into packaging material by blending or solvent casting technologies. Alternatively, adsorption or coating the packaging material can be used for the addition of essential oils. In addition, carriers in which essential oils are adsorbed or embedded can be used for the fabrication of small antibacterial bags continuously releasing the active compound in package headspace, or modified atmosphere packaging can be created by filling the entire packaging with essential oils in gaseous form [75]. The application of essential oils into hydrophilic materials by casting is mainly obtained by emulsification or homogenization techniques, which allow creating fine emulsion and after drying the lipid droplets remain embedded in the polymer matrix [66]. In addition, biopolymer-stabilized emulsions, also known as Pickering emulsions, are an interesting sustained delivery tool that was used for the same purpose [69,76,77]. They involve the stabilization of oil in water O/W or water in oil W/O emulsions by replacing surfactants with solid particles. A solid layer around the essential oils droplets prevents the contact with the aqueous phase, thus allowing higher coalescence stability, higher loading capacity, a more sustained release of the active agent and its protection from the external environment, particularly from oxidation [69,78].

Solutions of active packaging with essential oils and examples of applications can be found in recent reviews [66,75,79]. However, an important characteristic that should be taken into account is the interaction between essential oils and the packaging material, due to the alterations that the former can induce on the film. For instance, the addition of thyme essential oil to bio-nanocomposites made of whey protein isolate and cellulose nanofiber provided the decrease of water vapor permeability, the reduction of film transparency and the films obtained with thyme essential oil resulted as less strong and elastic than the control samples [73]. On the other hand, Mendes et al. [80] found that the addition of lemongrass essential oil by emulsification into formulations based on glycerol-plasticized cassava starch improved colorimetric attributes, thermal stability, barrier to moisture, and mechanical properties of film.

Active packaging has been developed using several natural extracts from plants and spices. Pure standards of natural antioxidants were used in several works to produce active films by different techniques. For instance, gallic acid and quercetin were added in polyvinyl alcohol (PVA) for the fabrication of active films by solvent casting [81], quercetin-starch-based complex were incorporated in chitosan-gelatin-based films [82], quercetin was adsorbed onto maize starch and calcium alginate aerogel through supercritical carbon dioxide adsorption [83], while α-tocopherol into polyethylene terephthalate/polypropylene (PET/PP) films was added by supercritical CO_2_ impregnation [84], and Poly(L-lactic acid) (PLLA)/antioxidant mixtures containing tocopherol and resveratrol were compression molded to films [85]. The antioxidant and antimicrobial properties of natural substances such as phenolic compounds are well-known in the literature [86,87,88]. Many plants, herbs and spices are considered to be potential antioxidant sources due to the rich amount of phenolic acids, carotenoids, and polyphenols in their extracts, which can be added to packaging materials, resulting in an extension of food shelf-life, as reviewed by Valdés et al. [89]. Free radical scavenging of phenolic compounds is mainly provided by two mechanisms, the former involves an H-atom transfer by the antioxidants, while the second a one-electron transfer, resulting in the formation of a nonreactive phenoxyl radical or a stable radical cation, respectively. The single antioxidant ability to inhibit oxidizing chain reactions depends on the molecular structure, conjugation, and resonance effects [90].

Very powerful antioxidants are also carotenoids. As reported by Stahl and Sies [91], they are most likely involved in the scavenging of singlet molecular oxygen, peroxyl radicals and they are effective deactivators of electronically excited sensitizer molecules, which are involved in the generation of radicals and singlet oxygen by physical quenching. Antimicrobial activity of phenolic compounds was explained by several mechanisms, such as aggregatory effect on all the bacterial cell, destabilization of cytoplasmic membrane, permeabilization of cell membrane, inhibition of extracellular microbial enzymes, deprivation of substrates required for microbial growth and direct action on microbial metabolism, depending on the subclass considered [86]. Several studies evaluated carotenoids antimicrobial activity against both Gram-positive and Gram-negative bacteria [92]. Recently, the antimicrobial activity of fucoxanthin, a carotenoid produced by brown algae and diatoms, was evaluated by Karpiński et al. [93]. However, some studies highlighted synergistic effects of natural compounds, able to increase both their antioxidant and antimicrobial activity when they are simultaneously present in mixtures or in extracts, compared to the same amounts of a single compounds [91,94,95,96]. For this reason, studies on active packaging dealing with the incorporation of natural extracts, rather than a single compound, in polymers for food applications are attracting more and more the attention of the scientific community. In addition, agrifood industry residues, such as spent coffee grounds [97], apple skins [98], grape seeds and skins [99], tomato waste [100], olive pomace [101], are still rich of antioxidant compounds, which can be recovered for active packaging purposes, allowing a waste valorization.

Examples of recent solution of active food packaging with natural extracts are reported in Table 3.

#### 3.6.3. Challenges and Solutions

One of the main disadvantages of natural antioxidants is related to their sensitivity to oxygen, heat and light, which can induce a loss of their activity. In particular, their thermo-sensitivity has implications both on their extraction, as well as in the packaging fabrication technique. Thanks to non-conventional processes such as supercritical-assisted extraction, ultrasound-assisted extraction, microwave-assisted extraction, solvent-free microwave-assisted extraction, pressurized liquid extraction, high pressure and temperature extraction, pulsed electric field-assisted extraction, whose efficiency and greener approach in the recovery the aforementioned compounds from natural sources was reviewed [109,110,111], the first problem can find effective solutions. The second issue can be faced performing the encapsulation of the active agent. By the encapsulation process, the active agent is entrapped in a carrier material and protected from external environment and stresses. Several advantages are related to the encapsulation, since solubility of the payload can be modulated, its delivery can be controlled, unpleasant odor and tastes can be masked, and the activity of the bioactive molecules can be extended, by choosing the suitable wall material [120]. In addition, the encapsulation of compounds for active food packaging can be used to improve the compatibility between the packaging polymer and the active agent, to increase its availability, to reduce changes in food sensorial properties or comply with the legal restriction limits [121].

Encapsulation of the active agent can be achieved by several techniques, as reported a recent reviews by Becerril et al. [121], Bahrami at al. [122] and Brandelli et al. [123]. For instance, Talón et al. [124] developed microparticles of soy lecithin, whey protein isolate, with and without and purified oleic acid, loaded with eugenol by spray drying, which were incorporated in corn starch films obtained by compression moulding. Zein electrospun nanofibers were used for the encapsulation of gallic acid [125], curcumin [126], rose hip seed oil [127], and β-carotene [128], while chitosan/PEO nanofibers containing microalgal phenolics were prepared to preserve their antibacterial activity [129]. Alternatively, zein microparticles were used for the encapsulation of the antioxidant luteolin [130], while the use of liposome nanoparticles was widely proved to be an efficient carrier for extract exhibiting antimicrobial and antioxidant activity [123,124,125]. Furthermore, antimicrobial activity of bacteriocins can be enhanced by encapsulation, which can help to reduce activity loss due to inactivation in complex food systems [57]. One of the most used and promising techniques involves the use of cyclodextrins, a family of cyclic oligomers of α-d-glucopyranose linked by α-1,4 glycosidic bonds, which were used for the preparation of active food packaging [126,127,128]. An upgrade of the encapsulation of active compounds by using cyclodextrins was given by cyclodextrin nanosponges, obtained by cross-linked cyclodextrin polymers nanostructured within a three dimensional network, which are stable in a wide range of pH and temperature, offering new perspectives to active packaging preparation techniques [131].

### 3.7. Phase Change Materials

**Figure 2:**
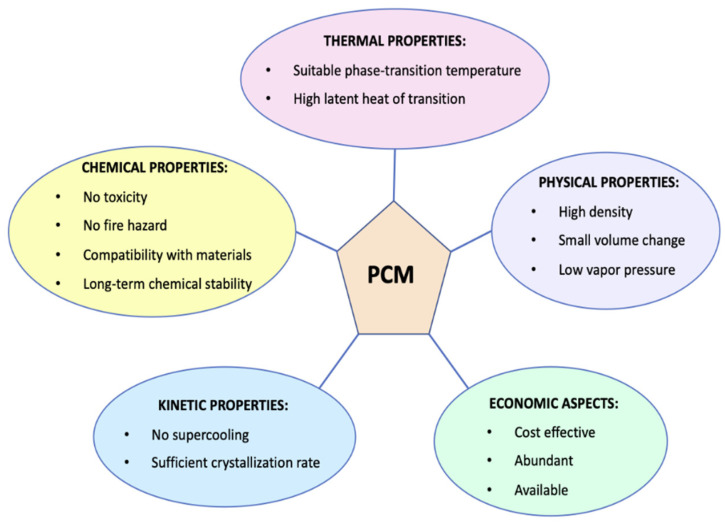
Main properties for ideal phase change material (PCM).

For many products, such as chilled foods, maintaining the cold chain is a crucial aspect to ensure food safety and quality. Traditional packaging materials are usually inadequate to respond to incorrect storage or transport conditions, showing low thermal buffering capacity [130,131]. Phase change materials (PCM) have aroused great interest as materials to be incorporated directly on the primary packaging, being able to prevent unwanted temperature changes and, therefore, to avoid or postpone consequent microbial, physical, and chemical food alterations [132]. A PCM is a substance that can undergo a phase transition at a specific temperature, absorbing or releasing latent heat, changing its phase from a solid state into a liquid or vice versa. PCMs have been used for several years, mainly for the storage of thermal energy [133], in the construction [134] and in the textile fields [135], and only recently they approached to the food sector [136]. Whatever the field of application, phase change materials must have some thermal, physical, kinetic properties and satisfy some economic aspects which are briefly reported in Figure 2 [130,136].

In a review by Sharma et al. [133] it is possible to find the main properties sought in the PCMs such as the melting point and latent heat of fusion values, as well as the typical measurement techniques of these parameters such as the differential scanning calorimeter (DSC) and differential thermal analysis (DTA). With regard to chemical properties, an interesting and complete work by Chandel et al. [137], compared all the categories of PCMs in terms of toxicity, health hazards and environmental effects.

Generally, PCMs can be classified into three groups of organic, inorganic, and eutectic materials [138]. The former can be further divided into paraffinic and non-paraffinic materials [139]. The most used paraffinic materials are paraffin waxes, consisting of a mixture of n-alkanes mainly with a linear chain (e.g., n-dodecane, n-tridecane etc.) [140]. They are chemically inert, non-toxic, non-corrosive, with small volume change and low vapor pressures, moreover they are safe and predictable [136]. On the other hand, paraffins usually have low thermal conductivity and are flammable in nature [133]. These problems can be solved by encapsulating the paraffins in an outer shell of a more stable supporting material, such as carbon nanotubes as investigated by Han et al. [141] for thermal management systems. Regarding the use of paraffins for applications in food packaging, some researchers have developed and optimized a smart packaging based on the encapsulation of dodecane into a biopolymeric matrix of zein using the uniaxial and coaxial electrospinning technique. The best results were obtained with coaxial configuration by adding a nucleating agent (tetracosane) to decrease the supercooling effect of the encapsulated [132].

However, the use of pure alkanes is quite expensive, therefore mixtures of alkanes with different molecular weights are usually used in order to regulate the melting temperature [142].

Non-paraffinic materials include fatty acids (myristic acid, capric acid, palmitic acid, lauric acid, stearic acid and their mixtures) [136], sugar alcohols (e.g., xylitol, D-sorbitol, D-mannitol) [137] and glycols (e.g., polyethylene glycol, triethylene glycol) [140,143]. In particular, fatty acids are among the most studied compounds for food applications due to the concurrent melting point, lack of supercooling, self-nucleating behavior, low volume change, high latent heat, chemical stability, and non-toxicity. The main problem is their high cost. They are also slightly corrosive in nature and have unpleasant odors [144]. In the work by Sathishkumar et al. [145] for example, the authors have investigated the use of palmitic acid as phase change material for application in food storage containers, obtaining better thermal energy storage than the same system without PCM. As for sugar alcohols, Palomo del Barrio et al. [146] have characterized several of these compounds (xylitol, adonitol, L-arabitol, erythritol, D-mannitol) for applications in heat storage, comparing their performance with those of the most used PCMs (paraffin waxes and salt hydrates). This study showed that the energy density provided by sugar alcohols and salt hydrates is much higher than that provided by paraffins and fatty acids.

Inorganic PCMs can be divided mainly into metallics (e.g., gallium, indium, bismuth) [133,140] and salt hydrates (e.g., potassium hydrogen phosphate hexahydrate, magnesium chloride hexahydrate) [133]. Materials belonging to this group have a higher phase change enthalpy than organic PCMs, but they show supercooling due to the poor nucleating ability, corrosion, phase segregation and phase separation [137]. Also in this case, these problems can be solved by encapsulating the inorganic PCMs as reported by Milián et al. [147]. While the problem of supercooling is treated in detail by Zahir et al. [148]. In this work, the authors discuss the main techniques to reduce the supercooling effect for the various types of PCMs, such as the addition of nucleating agents (e.g., carbon nanotubes, nonadditives). The authors also discuss the mitigation of phase separation and thermal cycling effects on supercooling. Salt hydrates have been used, for example, to improve the thermal performance of commercial freezers during the opening of the doors or the lack of electricity. The PCM used, a commercial salt hydrate composed of sodium nitrate dissolved in water, was able to keep the internal temperature of the freezer almost constant for 3 h in the absence of electricity, confirming the potential of these compounds in the transport and storage of perishable foods [149].

Finally, the eutectics consist of two or more low melting temperature components (e.g., tetradecane + octadecane, lauric + palmitic acid, water + polyacrylamide) [140]. The main advantage of eutectics respect other types of PCMs is that their melting points can be adjusted by combining different weight percentages of components [140]. Some researchers have characterized four types of micro/nano capsules composed of paraffins with different molecular weights encapsulated, via emulsion polymerization, using poly (methyl methacrylate) as shell material. The eutectic mixtures thus obtained showed good chemical and thermal stability and good thermal conductivity, resulting as suitable for food storage, as well as in the construction, textile, medical and electronic fields [150].

Most PCMs are liquid at room temperature, making difficult their direct incorporation into a package material. To overcome this issue, the encapsulation of PCMs in various polymeric matrices has been carried out, such as polystyrene, poly (butyl methacrylate), melamine-urea-formaldehyde [138], but also biodegradable polymers such as polycaprolactone [151], alginate [152], zein [132], gum Arabic and gelatin powder [153] have been investigated. In addition to preventing liquid leaks, the encapsulation techniques also serve to control the volume change during the phase transition [154], to protect the PCM from the external environment, in order to not compromise the thermal performance, and also to protect the external environment in the case of potentially corrosive PCM [155]. Moreover, the encapsulation allows obtaining a controlled release of thermal energy and an increase of the efficiency of heat transfer thanks to a larger area of heat transfer [147]. Generally, encapsulation techniques could be divided into three categories: chemical methodologies that include suspension polymerization [156], emulsion polymerization [157], in situ and interfacial polymerization [158,159]; physico-chemical methodologies, as coacervation [160], sol-gel encapsulation [161], supercritical CO_2_-assisted [162]; and physico-mechanical methodologies, like spray drying [163], electrospinning [158] and vacuum impregnation [164]. PCMs are a really interesting class of compounds to be integrated into the primary food packaging, but to date the applications in this sense are still few [132,165,166] compared to applications in refrigerated containers [145,149,151,167,168,169] or in the, already mentioned, construction and textile fields.

## 4. Intelligent Packaging

In the literature there are many definitions of the concept of intelligent packaging. European Commission defines intelligent packaging as “materials and articles that monitor the condition of packaged food or the environment surrounding the food” [10]. Intelligent packaging has also been defined as science and technology that uses the communication function to facilitate decision making to extend shelf-life, improve safety, guarantee quality, provide information, and warn of any problems by monitoring changes in the internal and external environment of the packages [6,170]. More in details, intelligent packaging is a packaging system that uses the internal (e.g., metabolites) or external (e.g., temperature) package environment as “information” to monitor the status of product quality, in order to improve not only product safety, but also to track the product for automatic identification and traceability [171].

The aim of intelligent packaging lies in its communication ability; the package can be considered the best companion of the product, since they constantly move together along the entire supply chain and therefore the package can, in principle, constantly communicate the conditions of the product [6,171]. The development of new intelligent packaging capable of continuously providing information on the conditions of both the food product and the packaging integrity, allows for a safer and more efficient supply chain, avoiding unnecessary transports and logistics and reducing food waste [7].

Products are regularly subjected to microbiological and chemical tests, which are carried out during production [172], but ensuring adequate control after delivery to the supermarket it is often not possible. Intelligent packaging can fill this gap [8]. Intelligent packaging can also contribute to improving ‘Hazard Analysis and Critical Control Point’ (HACCP) and ‘Quality Analysis and Critical Control Point’ (QACCP) methodologies, used to control, detect, prevent, reduce, and eliminate any possible criticism that could compromise the food product and its final quality [173].

The main technologies for intelligent packaging system are three: indicators, sensors, and data carriers [172]. Indicators and sensors have the main function of providing information relating to product quality, while the class of data carriers is more involved in the management of the supply chain logistics. These systems can be placed on the primary packaging, inside or outside, on the secondary or tertiary packaging [5].

### 4.1. Indicators

The main function of indicators is to transmit to the consumer information related to the presence or absence of a specific substance, to indicate whether a reaction is taking place between two or more components or to monitor the concentration of a certain substance. This information is translated into signals usually in the form of immediate visual changes (e.g., different color intensities or diffusion of a dye along a straight path) [6], providing qualitative or semiquantitative information. In most of the cases, the basic requirement of an indicator is that these changes in color or intensity are irreversible [174]. The indicators represent a very consistent class of intelligent packaging; the most used classification, also for this review, is based on the type of variable controlled, so they can be grouped into three macro categories: time-temperature indicators, freshness indicators, and gas indicators. For a more general classification, the indicators can be classified as external or internal, based on their positioning on the packaging [175].

#### 4.1.1. Time-Temperature Indicators

Temperature is a fundamental factor in monitoring and determining the shelf-life of a food product, especially for perishable products. One of the main challenges of the perishable food industry is to ensure the quality of food by protecting it from unwanted temperature fluctuations, a challenge that is not simple at all. Temperature monitoring and control along all the supply chain are essential to maintaining the quality and safety of perishable foods. Incorrect or uncontrolled temperature management can cause a large amount of food waste during production, as well as during the distribution chain. Studies report that poorly managed temperature in perishable food logistics can cause up to 35% loss of products [176]. To meet these needs, time-temperature indicators (TTI) provide valuable support.

Time-temperature indicators find application majorly for temperature-sensitive foods such as chilled and frozen products [174]. Furthermore, TTIs have also been applied to control the pasteurization and sterilization process [177]. The classification of TTI varies slightly from one author to another, the clearest grouping them into two categories: partial or full history indicators. A partial history indicator does not respond unless a certain predetermined threshold temperature is exceeded, and therefore identifies abusive temperature conditions, warning consumers of the potential survival of microorganisms and protein denaturation during, for example, freezing or defrosting [172,178]. A full history indicator responds continuously to all temperatures along the food supply chain, so as to provide a measure relating to the entire life of the product [5,179,180]. The TTIs currently available on the market, reported in Table 4, have operating mechanisms based on different principle that mainly include physical change or chemical reaction such as polymerization, melting or acid-based reaction, generally expressed through a visible response in the form of mechanical deformation, color development or color movement [181]. These types are the most common, but there is another class to mention, which is the enzymatic one, based on a biological response. In this case, TTI is sensitive to the change in biological activity of microorganisms, spores or enzymes [182]. The rate of change is directly proportional to the change in temperature similarly to the reactions responsible for the product deterioration. However, enzymatic TTIs still have limitations in terms of high cost and enzymatic instability. Some authors have developed an immobilized laccase-based TTI by electrospinning zein fiber to increase the stability of the enzyme. Immobilization onto solid carriers is the most used strategy to improve stability and fibrous membranes made by electrospinning have a great potential to be used for enzyme immobilization [183]. Usually, TTIs are made of small, self-adhesive labels attached onto containers or individual consumer packages, so as to be clearly visible and interpretable. These systems must represent time-temperature dependent changes in an easily measurable manner that can be quickly correlated with the extent of deterioration and the remaining shelf-life of the food [184].

#### 4.1.2. Freshness Indicators

Freshness indicators are intelligent devices that allow the monitoring of the quality of food products during storage and transport. Freshness decay can be due both to exposure to harmful conditions and to a shelf-life longer than that established. Freshness indicators, unlike temperature indicators, provide direct information on the quality of the product, analyzing the chemical reactions of food deterioration due to target microorganisms [171,175]. Changes in the concentration of metabolites such as glucose, organic acids, ethanol, carbon dioxide, biogenic amines, volatile nitrogen compounds or sulfur derivatives are indicators of microbial growth and their presence can therefore be exploited in the freshness indicators [185]. In most cases, freshness indicators are based on the use of dyes sensitive to pH variations caused by the deterioration of the product, which lead to the visible change in the color of the indicator. These indicators find applications in various products including fresh food, fruit, and seafood [174], but their marketing is still rare, especially in Europe, primarily for legislative issues related to substances that can be in contact with food. Table 5 reports some examples of freshness indicators present on the market and their main functioning.

In most cases, various polymeric matrices (e.g., polypropylene, nylon, or cellulose films) are impregnated with pH-sensitive dyes that are typically: bromocresol purple, bromocresol green, bromophenol blue, methyl red, and cresol red. In recent years, the use of natural dyes has also been studied. For instance, curcumin, grape peel, and beetroot extracts have been explored to detect the aging of cod flesh [186], or a system based on corn starch, chitosan and red cabbage extract was tested during fish spoilage [187].

Freshness indicators for seafood are based on the total volatile basic nitrogen content (TVB-N), as volatile amines, which are formed as the food spoils. Instead, hydrogen sulfide indicators can be used to determine the quality of meat products; in fact, hydrogen sulfide is correlated with the color of myoglobin, which is considered a quality attribute for meat products [188]. Most of these sensing mechanisms are based on color changing dyes, but penetration of the dyes into the package can affect the organoleptic properties of the packed food. To avoid this issue, some researchers have developed a freshness indicator consisting of a triple layer: a layer in contact with the food in non-woven high-density polyethylene, a layer containing a dye sensitive to pH variations, bromocresol green, immobilized with the binding polymer, and an external layer in low-density polyethylene. This indicator is able to detect chicken spoilage as a function of the change in concentration of volatile basic nitrogen, CO_2,_ and bacterial count, without migration of the dye inside the package, thus offering a safe packaging solution, simple and reliable [175]. Normally, freshness indicators are printed on the packaging film or are incorporated, in the form of labels, inside the packaging film, reacting with agents produced during the storage [189]. A common problem with the category of indicators is related to false negatives/positives. In the first case, there would be samples that seem safe but that are not, constituting a danger to the health of the consumer, while in the case of false positives the problem would be connected to the useless food waste induced by samples that seem dangerous or damaged, but which are actually healthy. In this sense, it would be necessary to develop standard protocols to accelerate the development of indicators, in particular those of freshness, also on an industrial scale [185].

#### 4.1.3. Gas Indicators

The activity of the food, the nature of the package, and the environmental conditions to which the package is subjected lead to the variation of the gas composition in the headspace of the package [6,190]. Gas indicators are used to monitor these changes and are also often used to evaluate the effectiveness of active packaging components (e.g., O_2_ and CO_2_ scavengers) [172] or to detect the occurrence of leaks across the packaging, and therefore to detect package integrity, which is an essential requirement, especially when the food is stored in a modified atmosphere packaging (MAP) [191]. The most well-known gas indicators are used to control oxygen and carbon dioxide concentrations [192]. Oxygen is responsible for the microbial and biochemical deterioration of food and is therefore removed from the packaging and replaced with gas such as nitrogen or using oxygen scavenger [193]. However, during transport and storage, oxygen may be able to penetrate the package due to poor sealing, defects, or damage. For this reason, the use of visible oxygen indicators becomes essential to quickly and easily verify the presence of oxygen inside the package, without using professional equipment or laboratory analysis [194]. The most commonly used oxygen indicator is a colorimetric indicator based on a redox dye, generally methylene blue and a reducing agent, such as glucose, in an alkaline solution such as sodium or potassium hydroxide [182,194]. If no oxygen is present, the glucose in the alkaline solution reduces the methylene blue which is, in this case, colorless. If oxygen is present in the monitored environment, the dye is oxidized and intensely colored. The reducing agent is sensitive to oxygen, therefore these indicators must be created and maintained in anaerobic conditions to avoid the rapid exhaustion of the reducing agent, due to the reaction with oxygen, and to the consequent malfunction of the indicator. Furthermore, there may be safety problems due to the contamination of the food product by the synthetic and, often harmful, chemical components used in the indicator [194]. A solution was proposed by Jang et al. [195], who developed an indicator by physically separating the components via a barrier. Activation of the indicator occurs by hand pressing and breaking the barrier, so components can come into contact. The rate of the color change is related to the oxygen concentration. In addition, the same authors have redesigned the indicator using natural organic compounds such as cysteine and laccase [194], thus finding a possible solution to the second issue mentioned.

The other predominant class of gas indicators is related to CO_2_. The microbial growth responsible for the deterioration of food usually begins immediately after packaging, and therefore there is a production of CO_2_ due to the metabolic activity of microorganisms. The rate of carbon dioxide production depends on the type of food, time, and conditions of storage, and on packaging materials [196]. Carbon dioxide is also used, in combination with oxygen and nitrogen, in protective atmosphere technology to inhibit microbial metabolism over time [197]. Again, most of the carbon dioxide indicators are colorimetric labels, whose color changes as a function of the pH of the product. Saliu and Della Pergola [198] have, for example, studied the performance of a colorimetric indicator composed of a mixture of lysine, polylysine and anthocyanins extracted from red cabbage, tested on cold-preserved foods such as poultry meat. The reversible reaction of amino group of lysine in presence of CO_2_, leads to the formation of the corresponding carbamic acid derivative, and to a consequent variation in the pH of the solution, which is shown by a significant variation in the color of the anthocyanin dye. In recent years, anthocyanins have attracted the attention of many researchers who have investigated their behavior as natural dyes, in combination with natural-based polymer matrices, for the production of safe, non-toxic, biodegradable, and suitable colorimetric indicators to be use in the food sector [199,200,201].

Gas indicators, mainly in the form of labels or printed directly on the packaging films, are positioned inside the package to monitor changes in the internal gas composition, thus providing a strategy for monitoring the quality and safety of food products [6].

Table 6 reports some examples of gas indicators present on the market.

### 4.2. Sensors

A sensor is a device that responds to a chemical, biological, or physical property by providing a quantifiable signal proportional to the measurement [172]. The most popular traditional sensors are designed to measure temperature, humidity, pH, and light exposure [7]. In addition to these, the need to monitor food quality and packaging integrity has led researchers to place a growing interest in applying disposable and advanced sensors (such as edible sensors) for intelligent packaging. Sensor usually consists of four main components: the first part is a receptor (the sensitive part of the sensor), which generally consists of a selective coating that acts as a sampling area that is able to detect the presence, activity, composition, or the concentration of specific chemical analyte by surface adsorption, with consequent modification of a certain property of the coating [7]. This change is typically detected by the transducer, which is the second part of the sensor, converting the signal change into an output signal. If the transducer requires external power for measurement, the transducer is called “active”, if not, it is called “passive” [7,173]. Finally, the sensor is completed by a part of electronics for signal processing and a signal display unit [172].

The integration of these disposable into food packaging is not simple or immediate; in fact, there are very few products currently on the market, reported in Table 7. This class of intelligent devices has yet to overcome several obstacles before it can reach large-scale marketing. In particular, extensive research efforts are still needed to study the potentially dangerous effects of the chemical and biological components present in the sensor on foods, to avoid any migration of these components, and also to avoid potential changes in taste and consistency of the food products [202]. Disposable sensors for food packaging should also satisfy the following requirements: selectivity for the target species; sensitivity to changes in target species concentrations; quick response times; small size (miniaturization); low construction cost [172]; use inexpensive, sustainable or biodegradable materials; be simple to use; operate without or with an affordable, portable instrument [203]. Furthermore, the approach to sensors is made difficult by the fact that the terms indicators and sensors are often used indiscriminately. In fact, in the literature, the term sensor is often referred to a device capable of detecting a specific analyte, a definition which includes both sensors and indicators. Actually, they represent two very distinct classes of devices [202]. An indicator, as reported in the previous chapter, checks a condition, and displays it directly by providing qualitative or semi-quantitative information through a visible change that is easy to interpret. On the contrary, a sensor can provide quantitative results and must send the transduced signal to an electronic or logical module for the interpretation of the information [204]. Sensors intended for food packaging applications can be classified into two macro categories: chemical sensors and biosensors. Each of these two categories can in turn be divided into subclasses based on the method for signal transduction: electrochemical; optical; mechanical; magnetic; thermometric, and microgravimetric.

Certainly less interesting for food application, mechanical sensors detect physical changes due to stress: they are mainly used to measure physical quantities, such as force, acceleration, pressure, and flow [205]. Microgravimetry is used to measure mass changes and offers a possible approach for unlabeled detection of biomolecules [206]. Thermometric sensing devices transduce temperature change into an electrical signal and include: thermocouples, resistance thermometers, thermistors, and diodes [207]. Magnetic methods are still rarely used in disposable sensing devices [208]. Instead, some of the most recent chemical sensors or biosensors for food applications are based exclusively on the electrochemical [209,210,211] and optical transduction methods [212,213]. Therefore, these two types of devices will be discussed in the following paragraphs, in which examples of applications have been reported in order to try to provide a clear and complete picture of this complex class of devices.

This category of devices is usually positioned inside the packaging, separated from food product. The future developments aim to integrate the sensors into the package itself through printed electronics technology [214,215], using electrically functional inks on flexible substrates (polyamide, PET, transparent conductive polyester, steel, paper) [7]. This technology has numerous advantages, including the possibility of having lightweight, thin, foldable, portable, and selective sensors [7,173]. Moreover, the initial problem of the low performance that these devices had shown is finding a solution in the use of nanomaterials [216,217].

Finally, due to the trend of recent years, it will be possible to find sensors also in contact with food thanks to the formulation of edible sensors, a very particular category of devices that will be addressed at the end of this chapter.

#### 4.2.1. Chemical Sensors

Chemical sensors are the most suitable devices for the realization of intelligent food packaging thanks to the receptor, which is chemically capable of detecting the presence, activity, composition and concentration of a specific chemical molecule, such as volatile organic compounds (VOCs) and gas molecules (H_2_, CO, NO_2_, CO_2_, H_2_S, etc.), which are primarily responsible for food spoilage [7,173], especially for meat, fish, fruit and vegetable products. In fact, the composition of gas within food packaging changes due to the activity of the food products, the gas permeability of the packaging material, and the environmental conditions. These changes are directly related to the shelf life, quality, and safety of packaged food [190]. For this reason, most of the chemical sensors that are being studied are gas sensors based, as mentioned, on electrochemical or optical signal transduction methods. In general, these sensors could be used to replace traditional analysis performed with fixed or portable instruments, such as gas chromatography mass spectrometer or gas analyzers that require breakage of package, do not allow real time measurements and on-line control, and are available for small-scale use. However, chemical sensors currently on the market, are still too large and rigid to be integrated into the packaging, not sufficiently selective and still require a high power (e.g., high operating temperature) [173]. Furthermore, their marketing in the food sector has so far been hindered by high development and production costs [218]. However, interesting works can already be found in the literature [219,220,221,222], the number of which is constantly growing, confirming the interest of researchers in this field.

#### 4.2.2. Electrochemical-Based Sensors

Regarding electrochemical sensors, the transduction element is represented by an electrode. A typical electrochemical sensor consists of three main electrodes: working electrode, counter, and reference electrode, which are all connected to a potentiostat device. By applying a voltage via the potentiostat, a redox reaction occurs at the electrode/analyte interface, which thus causes an electrons transfer between the electrode and the electroactive species that generates a current proportional to the concentration of the analyte [172]. The most important conventional electrochemical techniques are potentiometry, amperometry, voltammetry and conductometry, depending on the signal supplied by the transducer [219]. Established systems for chemical detection, such as metal oxide semiconductor field-effect transistors (MOSFETs), piezoelectric crystal sensors, amperometric oxygen sensors, organic conducting polymers, and potentiometric carbon dioxide sensors, are subject to cross-sensitivity to carbon dioxide and hydrogen sulfide, contamination of sensor membranes, and consumption of the analyte and, in most cases, these systems involve destructive analyses of packages [172].

The innovation for these devices starts from the choice of the material for the working electrode which is one of the most important factors in the design of an electrochemical sensor. Commonly used electrode materials are inert metals, semiconductor metal oxides, such as zinc oxide, and carbon-based materials. Certainly, a first step toward innovation is to start paying increasing attention to the production of biodegradable electrodes, using for example activated charcoal, magnesium, or melanin, using the biomedical field as a model, in order to reduce the environmental impact and the costs of disposable detection devices [203]. However, the combination of good and stable electrical performance with chemical degradation and high mechanical deformation still remains difficult because electrical performance is limited [220]. However, even more noteworthy is the fact that in recent years there has been an increase in studies regarding the use of nanomaterials in detection, especially in gas detection. The interest is particularly focused on carbon nanomaterials such as nanoparticles (carbon and fullerenes), graphene, graphite, nanofibers, and nanotubes. Carbon nanomaterials offer a high specific surface area and therefore exhibit excellent detection sensitivity. Together with their excellent electrical properties and mechanical performance, such as light-weight and high flexibility, even at low temperatures, they are suitable for application in chemical sensors, both as a receptor and transducer [173,202]. Carbon nanotubes and graphene exhibit concentration-dependent resistivity changes due to adsorption of gas molecules, so they are good candidates for gas sensors. In particular, thanks to its two-dimensional shape, each graphene atom could be considered to be a surface atom. In this way, each atom site could be involved in gas interaction, greatly increasing the detection capacity, with the detection of a single molecule [173]. Fullerenes are extremely strong molecules, capable of resisting high pressure (over 3000 atm), so they are suitable as receptors. Carbon nanofibers are extremely pure materials, suitable for use as receptors thanks to the high mechanical strength and high geometrical surface [173]. Although carbon nanomaterials are very promising for the development of a new generation of miniaturized chemical sensors with high performance, their development for food packaging applications is still far away due to some important technological limitations. First of all, the presence of contaminants on the surface must be absolutely avoided. This problem could be solved by functionalization of the carbon nanomaterials surface with specific chemical or biological molecules. Furthermore, the cost must be lowered and the production methods must be industrial scaled [7,173]. In this context, some authors have successfully studied different carbon nanotube functionalization methods for the electrochemical detection of bisphenol-A, a potentially harmful organic compound that can pass from plastic containers to food [211]. Other authors have fabricated a new electrochemical sensor to detect thiamphenicol antibiotic residues in milk. To increase the sensitivity of the sensor, the authors modified the electrode by simultaneously using carbon nanotubes and gold nanoparticles [221].

Another class of compounds that needs to be monitored is that of nitrite, potentially present in foods such as sausages and pickles that could be detected electrochemically. N-doped graphene quantum dots decorated N-doped carbon nanofibers membrane were used. Incorporation of N-doped graphene quantum dots increased the electron transfer rate and N-doped carbon nanofibers provided greater electrical conductivity and large electroactive area and free-standing film structure, making the composite appropriate for the electrochemical sensor. This quantum dots-based nanofiber sensor has shown high performance for nitrite sensing with a low detection limit, a parameter that must always be considered, in addition to high selectivity and excellent reproducibility [222].

Finally, great interest is turned toward a type of gas sensors capable of detecting odors that are an indication of food spoilage. In fact, food spoilage processes are associated with the presence of certain odors. Studies show that the biogenic amines, such as tyramine, tryptamine, putrescine, cadaverine, were significantly related to traditional quality indices in meat products. Since individual chemical sensors are designed to be highly selective for a limited selection of specific compounds, a system capable of detecting each compound present in a given odor is required. One such system is called a nose system or electronic nose. It is sometimes titled as electronic tongue if the sensors array is designed for liquids [223]. The idea was introduced in the 1990s and, since then, a huge amount of work has been done in this field [224]. A nose system is able to imitate or exceed the human sense of smell or taste by generating a unique response for each odor/smell. A nose system is composed of a one- or two-dimensional array of chemical sensors, but also biosensors, which allow the recognition of simple or complex flavors [7]. Main electronic nose (E-nose) applications in food are fermentation [225], process monitoring [226], quality evaluation [227], ripening and shelf-life investigation [228], and also authenticity and product traceability [229]. These types of sensors can be both chemical and biosensors and can be based on both the electrochemical and optical transduction method.

For example, the response of the E-nose has been found to be consistent in the quality evaluation of fresh yellow din tuna and vacuum-packaged beef [178]. In an interesting study, the applicability of E-nose for quality control of MAP broiler chicken cuts in different temperature regimes using 24 chemical sensors was evaluated. The results of the electronic nose were compared with those obtained from the microbiological, sensory and headspace gas chromatography analyzes, indicating that the electronic nose was also able to detect the first signal of spoilage compared to other techniques [230].

#### 4.2.3. Optical-Based Sensors

Optical sensors generate an optical signal (color, fluorescence or chemiluminescence), or cause a change in the optical properties of the system. The optical signal produced can be observed with the naked eye or can be measured by a photodetector that converts the optical signals into measurable electrical signals. Compared to sensors based on electrical transducers, sensors based on optical transducers do not need electrical power supply and can be powered and read out from a distance by using UV, visible, or IR light. Optical methods have two main drawbacks: susceptibility to environmental interference and the use of fragile and sometimes expensive materials that require careful handling (e.g., optic fiber). The advantages of these techniques are fast response, sensitivity, reliability, and they are mostly non-destructive. The most important materials for optical detection include dyes, gold and silver nanoparticles, photonic crystal, and graphene nanomaterials [203]. In particular, oxygen sensors based on the detection of luminescent or fluorescent signals have proven to be more sensitive and more accurate in quantitative measurements than visual oxygen indicators, thus providing a valid alternative to the latter [172]. Fluorescence-based oxygen sensor involves coating the device, such as an optical fiber, with a dye-polymer, obtained by immobilizing a fluorescent dye in a solid polymer matrix via, for example, encapsulation. This sensor detects the presence of molecular oxygen which, if present in the packaging, gradually quenches the luminescence. The oxygen concentration is therefore related to the degree of alteration of the luminescence intensity. The process is clean, does not lead to the consumption of dye or oxygen and does not produce by-products [231]. Materials for oxygen sensors must have fluorescent characteristics that allow for the construction of measuring devices that are as simple as possible. The most suitable fluorescence and phosphorescence dyes for detecting oxygen in food packaging are those with a lifetime in the microsecond range. They must also have an adequate luminescence intensity, longwave emission bands and good photostability. Dyes such as platinum-porphyrins or ruthenium-metalloporphyrins combined with polystyrene as polymer matrix appear to offer the greatest potential [232,233]. The polymer matrix must be compatible with the dye, allow oxygen permeability and, more generally, have good mechanical resistance. Microporous support materials seem to possess the necessary requirements to be used in the detection sensors for food packaging [234]. Instead, polymers such as polyamide, polyethylene terephthalate and polyvinyl chloride, have good gas barrier properties but are not suitable for oxygen detection since oxygen quenching is slow in these polymers, while plasticized polymers have proven unsuitable due to toxicity problems associated with potential migration of compounds into food products [218].

O2xyDot^®^ is, for example, one of the few fluorescence-based oxygen sensors currently on the market, which is developed by OxySense^®^, as reported in Table 7. This sensor is designed to be placed inside the package and to be read from outside without compromising the packaging structure. O2xyDot^®^ can be used both in the headspace of the package and in contact with the products, such as for example, for liquid products. As can be seen from the name, this sensor is composed of a dot which, once illuminated by a pulsed blue light from a light emitting diode (LED), absorbs the blue light and emits a red light which is detected by a photodetector that allows measuring the levels of oxygen in the package according to the duration of the fluorescence. The presence of oxygen quenches the fluorescence of the dye. In fact, the energy is transferred from the excited fluorescent dye to the oxygen molecule, during a collision, thus reducing the intensity of emission and the lifetime of the fluorescence of the dye [231]. This type of sensor is perfect to be used to check the quality of products such as meat packaged in a modified atmosphere and also liquid products. There are also good expectations for the detection of carbon dioxide. For example, some researchers have developed a solid-state polymeric optochemical sensor to detect carbon dioxide, based on the forster resonance energy transfer mechanism, using the dye (phosphorescent Pt-porphyrin) and α-naphtholphthalein, a phthalein dye, as the indicators. The developed sensor was also characterized and tested for toxicity due to migration of dyes [175,187].

A sensor based on the same principle was also studied, with a different pH indicator dye (Sudan III co-immobilized in the sol-gel matrix-ruthenium polypyridyl complex), which was able to detect even low concentrations of CO_2_ with accuracy and rapidity. However, both the sensors described have shown cross-sensitivity with O_2_ detection, therefore further studies are necessary [175].

An interesting class of devices that have been developed for very selective gas concentration detection in the headspace of food packaging are silicon-based optical transducers composed of optical circuits integrated in silicon semiconductor material [235].

In particular, silicon-on-insulator (SOI) micro-ring resonators (MR) are very small and efficient optical transducers that have high refractive index sensitivity. They are based on the optical detection of small changes in the refractive index: near-infrared light coming from a remote light source propagates through the SOI MR, thus detecting the variations of the refractive index in the receptor. Therefore, this results in a frequency shift of the light leaving the SOI. A chemical sensor of this type has been studied for the detection of ammonia gas. The sensor was coated with a nanoporous amorphous silica-alumina film and demonstrated sensitive and reversible NH_3_ detection in real time at room temperature [7,236]. Other authors have studied a very small silicon photonic-based chemical sensor to measure VOCs and CO_2_ concentrations in the headspace of food packaging [7].

In general, this class of sensors can present problems related to noise sensitivity, low detection sensitivity, and high operating costs, mainly due to the use of infrared lasers and detectors, necessary for reading the sensor. The main advantages, on the other hand, are low cost of production and the possibility of using the same instruments and methods usually used for the production of conventional silicon semiconductors thus allowing a large-scale production [173].

#### 4.2.4. Biosensors

Biosensors differ from chemical sensors mainly in the receptor. While in chemical sensors the receptor is a chemical compound, in biosensors it is made of organic or biological material, such as DNA, RNA, enzymes, antibodies, antigens, microbes, hormones and nucleic acids [6,7,172]. Biosensors can be applied to identify, record and measure allergens and analytes such as sugars, amino acids, alcohols, lipids, pathogens, etc. [7] and target metabolites produced by biochemical reactions occurring in the food degradation process [173].

The main challenges for this class of sensors are: immobilization of biological components in the receptor, for example through robust attachment methods such as electro-deposition, prevent denaturation, or degradation of the biological components; obviously, it is necessary to completely avoid the possible hazardous effects due to the migration of biological components on food [7,173].

Again, the transducer can be electrochemical or optical-based [190]. Among the various biosensors reported in the literature, the electrochemical ones are the most widely studied.

#### 4.2.5. Electrochemical-Based Biosensors

Electrochemical biosensors combine the advantages of analytical electrochemical techniques and the advantages of the specificity of biological recognition processes. The goal of a biosensor is to produce biologically, after immobilization of a biospecific reagent on an electrode, a measurable electrical signal, related to the concentration of a specific analyte [237]. The progress that has reached this category of sensors is certainly due to biomedical studies, from which derives for example the first biosensor with enzyme electrode for the detection of glucose in the blood. Enzyme-based biosensors dominate the market and are mostly based on electrochemical transduction systems [238]. Today, even in the food packaging field, there are works dealing with the detection of glucose in various beverages. For example, some authors have studied an electrochemical biosensor based on an electrospun nylon-6 nanofibrous membrane, functionalized with glucose oxidase enzyme, immobilized on the membrane by chemical cross-linking via glutaraldehyde and bovine serum albumin. This biosensor has proven effective in glucose detection [175].

The detection of glucose levels can indicate the presence of deterioration, for example, of meat. Glucose detection was also possible via a glucose sensor that used a gold electrode modified using L-cysteine and a nanogold solution, coated with a polyglutamate-glucose oxidase complex dropped on the modified electrode [239]. Moreover, numerous electrochemical biosensors have been used for the detection of bacteria and parasites. The achievement of sensitive detection limits for microbes was supported by incorporating nanomaterials such as gold nanoparticles, carbon nanotubes and graphene oxide, which was discussed in the paragraph relating to chemical sensors, for example for detection of the most common food pathogen *Salmonella typhimurium* in pork [240].

#### 4.2.6. Optical-Based Biosensors

Food contamination is a very serious problem that can occur by pathogenic bacteria such as *Escherichia coli*, *Salmonella typhimurium*, *Staphylococcus aureus*, *Bacillus cereus*, *Streptococci*, etc., which can cause several disorders and are responsible for about 90% of all food-borne illnesses [241]. Therefore, food safety is one of the main objectives of both legislation and packaging industry. For example, Toxin Guard^TM^ (by Toxin Alert) is a visual biosensor used to detect pathogens that can contaminate food, including *Salmonella*, *E. coli*, *Listeria and Campylobacter*, *based on* antibody-antigen reactions. In the presence of pathogenic bacteria, the bacterial toxin is bound to the antibodies and immobilized on a thin layer of flexible film, in this case polyethylene, producing a clear change in the color of the biosensor [8,172]. Based on the same principle, commercially available Flex Alert biosensor have been developed against *E. coli* O157, *Salmonella* spp., *Listeria* spp., and aflatoxins, especially for dried fruit [172].

The bottleneck of these sensors is the difficulty of integrating them into food packaging. A solution seems to be offered by molecularly imprinted polymer biosensors, a technology that allows the production of elements for the recognition of analyte. First, a solution is created between the analyte and the polymer, then the solution is polymerized. At this point, after the polymer has formed, the molecules of analyte are removed, leaving cavities with a specific shape for each molecule in the polymer, in which each molecule can be identified if detected. This technique has been used, for example, for the development of a biosensor for food spoilage, obtained using a polymer containing a polyazamacrocyclic transition metal complex. The complex selectively binds to biogenic amines, such as cadaverine, putrescine and histamine, which are released by microorganisms during deterioration process. The polymer undergoes a detectable color change upon exposure to biogenic amine, thus indicating that food spoilage has occurred [239].

#### 4.2.7. Edible Sensors

One type of sensors that is expected to have great success in the field of intelligent food packaging is that of edible sensors for detecting food deterioration, made using only natural and biodegradable materials without negative or dangerous effects on humans even in the long term. For example, a sensor has been developed consisting of a pectin matrix containing a red cabbage extract as a colorimetric indicator. Pectin is a natural polysaccharide commercially extracted from citrus fruit and apples and is widely used in the food industry to improve the gelation of food products. The red cabbage extract contains a significant quantitative of anthocyanins, in particular the derivatives of cyanidin glycosides. Anthocyanins are known pigments with the ability to change color when exposed to pH changes and are able to detect the presence of amines [242].

Other authors have developed an edible film based on gelatin, gellan gum, and red radish anthocyanin extract, sensitive to gas and showing a change in color from orange-red to yellow, with a change in pH in the range of 2–12. This edible sensor has been successfully applied in real time detection of milk deterioration, detecting gas produced by anaerobic bacteria, and fish spoilage, detecting gases such as ammonia, trimethylamine, and dimethylamine, produced by the decomposition of proteins by bacteria and enzymes, causing a change in the color of the film [175].

The use of genipin, a natural iridoid, has also been successfully studied as a dual colorimetric sensor for both oxygen detection and biogenic amines. Genipin was immobilized in edible calcium alginate microspheres [243]. These and other similar works will certainly pave the way for the development of sensors based on natural compounds capable of detecting the most common analytes for food products, also using biodegradable matrices, non-toxic and biocompatible with food. In this way all the advantages deriving from the use of sensors in food packaging could be combined, guaranteeing quality and safe products, not only for human consumption but also for the environment.

### 4.3. Data Carriers

Data carrier devices represent a type of intelligent packaging with a different function than the two classes described in the previous sections. In fact, they do not provide any information on the quality status of packaged foods, but are an important support for automated traceability, theft prevention or protection against counterfeiting [8]. Traceability, in particular, allows improving food safety and to achieve a better market for consumers since it is easy to trace the complete history of the package [175]. The most important data carrier devices in the food packaging sector are barcode labels and radio frequency identification systems (RFID tags). These types of devices are mainly placed onto tertiary packaging (e.g., containers, pallets, etc.) [172], to be legible throughout the all supply chain.

Barcodes have been widely used since the beginning in large-scale retail trade to facilitate inventory control, stock reordering, and checkout [244]. One-dimensional barcodes have been developed for first but with limited data storage capacity [180]. Subsequently, Reduced Space Symbology (RSS) barcodes were developed to encode more data in a smaller space. Finally, the latest Quick Response (QR) 2-D barcode allows storing even more data using four different encoding modes: numeric, alphanumeric, byte/binary and kanji, the latter referring to logographic Chinese characters. Reading 2-D barcode symbologies require a scanning device capable of reading simultaneous in two dimensions, both vertically and horizontally [172].

However, the most advanced data carrier devices are represented by radio frequency identification systems. An RFID system is composed of three main elements: a tag consisting of a microchip connected to a small antenna; a reader that emits radio signals and receives responses from the tag in return; and middleware (a local network, web server, etc.) that connects RFID hardware and enterprise applications [245,246]. The main features that make RFID technology unique are: the high number of different codes that can be stored in the tag and the ability to transfer and communicate information even over long distances, thus improving automatic product identification and traceability operations [247]. Commercially available RFID technologies are reported in Table 8. Currently, RFID technology includes two types of tags: active and passive tags. The main differences lie in the fact that active tags have their own power supply (battery), have a transmission distance of 20 to 100 m and can communicate with the reader at any time, are more expensive and larger than passive tags. Passive tags, on the other hand, acquire the power from the external radio frequency communication, have a lower transmission distances, from a few centimeters up to 10 m, are activated when they are within the radius of a RFID reader, are less expensive and smaller than the active tags [172]. However, RFID technology should not be seen as a total replacement of barcodes, but these two technologies can continue to be used in combination as well.

## 5. Conclusions

This review underlines the effectiveness of active and intelligent packaging demonstrated by the large number of scientific studies discussed. The advantages of smart packaging application in terms of safety and logistics, as well as marketing, indicate that intelligent packaging and active packaging could become part of the industry, and may even dominate it in a few years. However, a gap still exists between research activities and laboratory solutions and real market products. In this sector, a greater customization of the packaging system is needed; indeed, food products are very complex systems and packaging parameters are highly product-specific. Thus, to achieve an optimal activity or capacity of the desired smart packaging system, product-tailored concepts have to be introduced. Research efforts should be directed to fill this gap, trying to provide specific solutions developed and tested for selected product categories. A successful collaboration between research institutes and industry, including development, legislative and commercial functions, is required to overcome these challenges. Working in this direction will accelerate the commercial adoption of this innovative packaging system.

## Figures and Tables

**Table 1:** Commercially available food active packaging.

Commercial Name	Principle	Application	Materials and Forms
Activ-Film^TM^ (www.csptechnologies.com)	Moisture absorber	Fruit and vegetables	Low-density polyethylene (LDPE) film
Tenderpac^®^ (www.sealpacinternational.com)	Moisture absorber	Meat products	Polyethylene terephthalate (PET) tray
PEAKfresh^TM^ (www.peakfresh.com)	Ethylene scavenger	Fruit and vegetables	Film impregnated with a natural mineral
BIOPAC (www.biopac.com.au)	Ethylene scavenger	Fresh products	Sachet in porous material mixed with potassium permanganate
ATCO^®^ (www.emcotechnologies.co.uk)	Carbon dioxide absorber	Fresh products	Film—bags
SANDRY^®^ (www.hengsan.com)	Carbon dioxide absorber	Fruit, coffee, fermented food	Sachets
McAirlaid’s CO_2_Pad (www.mcairlaids.net)	Carbon dioxide emitter	Fish, meat and fruit products	Cellulose-based pads
FreshPax^®^ (www.multisorb.com)	Carbon dioxide emitter	Processed and pre-cooked food	Packets and films realize with food grade materials
Celox^TM^ (www.grace.com)	Oxygen scavenger	Beverages	Cans sealants and closure coatings
ZERO_2_ (www.ipl-plastics.com)	Oxygen scavenger	Fresh products	Multilayer film fused to injection-molded containers
Biomaster^®^ (www.biomasterprotected.com)	Antimicrobial	Chilled and frozen products	Cool bags
Food-touch^®^ (www.microbeguard.com)	Antimicrobial	All food products	Various forms of paper products
ATOX (www.artibal.com)	Antioxidant	Cereal products	Film coating containing oregano essential oils
Pure Temp (www.puretemp.com)	Phase Change Materials	Frozen food, cold storage	Palm oil, coconut oil and soybean oil-based
Green Box (www.greenbox.it)	Phase Change Materials	Perishable products	Vegetable oil-based

**Table 2:** Main features of commercially available systems working as moisture regulators.

Structures		Desiccants	
		Inorganics-Based	Organic-Based
Sachets	systems where moisture scavenger materials are enclosed into a small porous bag.	bentonite calcium chloride calcium oxide calcium sulfate molecular sieves natural clays silica gel	cellulose fructose modified starch sorbitol
Pads, blankets, sheets, trays:	mostly composed of porous materials, foamed and perforated sheets and moisture superabsorbent materials.		
Labels	systems composed of adhesive dessiccant labels, suitable for low-level humidity systems.		

**Table 3:** Examples of packaging with natural extracts as active agents.

Active Agent	Extract Production	Polymer	Active Packaging Production Technique	Food	References
Rosemary extract	Solid-liquid extraction (L/S = 10 mL/g, 50 °C, 60 min, solvent: water)	Cassava starch film	Solvent casting	Simulants (water and ethanol 95%)	[102]
Green tea extract (inorganic capsules)		Polyethylene		Meat	[103]
Garlic extract		Polyethylene/polyethylene; Polyethylene/ethylene-vinyl alcohol copolymer; Polyethylene/zein	Corona treatment + spreading	Bread	[67]
Propolis extract	Solid-liquid extraction (50 °C, 24 h, solvent: 30% ethanol aqueous solution)	Chitosan	Solvent casting		[104]
Marjoram essential oil (encapsulated in nanoemulsion and Pickering emulsion)	Hydrodistillation 4 h	Pectin	Casting	Simulant (ethanol 95%)	[76]
Mint leaves and pomegranate peel extract	Reflux extraction, L/S = 10 mL/g, 1 h, distilled water	Polyvinilalcohol/chitosan	Casting	Simulant (distilled water)	[105]
Murta fruit extract	Solid-liquid extraction, 40 °C, 3 h, ethanol 50%	Methyl cellulose	Solution-extension-evaporation (“casting”)		[106]
Z. clinopodioides leave extract, ethanolic grape seed extract	Hydrodistillation, 3.5 h (for Z. clinopodioides leave extract)	Chitosan, gelatin	Casting		[107]
Grapefruit peel and mint leaves extracts	Reflux Extraction, water, 1 h, L/S = 10 mL/g	Guar gum/chitosan/ polyvinyl alcohol	Casting		[108]
Rice straw extract	Solid-liquid extraction, L/S = 10 mL/g, 1 h, room temperature, water	Potato starch	Compression-molding		[109]
Green tea and basil extracts	Infusion (L/S = 100 mL/3 g, 100 °C, 40 min)	Cassava starch	Casting	Simulant (water)	[110]
*Olea europea* leaf extract	Enhanced solvent extraction (solvent: CO_2_ +50% ethanol, 120 and 200 bar, 55 and 80 °C, total flow of 10 g/min, 2 h)	Polyethylene terephthalate/polypropylene	Supercritical solvent impregnation	Simulants (distilled water, 3% acetic acid, 95% ethanol)	[111]
*Thymus vulgaris* L extract	Supercritical extraction	Polylactic acid/ polycaprolactone	Solvent casting+ Supercritical solvent impregnation		[112]
*Allium ursinum* L. (wild garlic) extract	Ultrasound-assisted extraction (20.06 W/L, 80 °C, 80 min, solvent-to-solid ratio = 5 g/g, solvent: 70% ethanol-in-water solution)	Polylactic acid	Electrospinning + annealing		[113]
Tomato by-product extract	Ultrasound-assisted extraction (ethanol 98%, solvent-to solid ratio 5 mL/g, 30 min)	polyvinyl alcohol/chitosan	Solvent casting		[114]
*Persicaria minor* extract	Solid-liquid extraction (aqueous solvent containing 75% ethanol at a ratio 1:20 (*w*/*v*), under shaking at 150 rpm, room temperature, 24 h).	Semi-refined carrageenan powder	Solvent casting	Meat Patties	[115]
*Aloe debrana* and papaya leaves extract	Aloe gel extraction and homogenization: solvent-free extraction by screw press (for papaya leaves)	Gelatin	Solvent casting		[116]
Apple pomace extract	Soxhlet extraction (ethanol:water solution 80:20 *v*/*v*, 120 min, solvent-to-solid ratio 10 mL/g)	Bacterial cellulose-based nanopapers coated with a hydrophobic medium chain-length polyhydroxyalkanoate	Solvent casting		[117]
Pumpkin residue extract and oregano essential oil	Solid-liquid extraction/homogeneization (ethanol, solvent-to-solid ratio 20 mL/5 g 10 min)	Cassava starch	Solvent casting	Ground beef	[118]
mango peels extract	Maceration (ethanol, solvent-to-solid ratio 10 mL/g, 24 h)	Fish gelatin	Solvent casting		[119]

**Table 4:** Commercially available time-temperature indicators (TTI) indicators.

Commercial Name	Application	Principle
3M^TM^ MonitorMark^®^ (www.3m.com)	Backery, beverage, meat	Self-adhesive pad for easy attachment to secondary packaging to monitor temperature exposure, not product quality. The pad containing a blue dyed fatty acid ester inside a carrier substance. The dye remains inside the pad until the carrier undergoes a phase change due to temperature exposure above the response temperature, then the dye diffuses along a wick and the distance the dye has migrated along the track is measured as response
Fresh-Check^®^ Temperature Intelligence^TM^ (www.fresh-check.com)	All fresh products	Self-adhesive device based on solid state polymerization reaction resulting in highly colored polymer. As the active center exposed to the temperature over time it gradually changes color to show the freshness of the food. The active center circle darkens irreversibly
Insignia Deli Intelligent Labels^TM^ (www.insigniatechnologies.com)	Chilled products	Color change accelerates with fluctuation or change in pre-calibrated temperature range
OnVu^TM^ (www.packworld.com)	Meat, fish and dairy products	It is composed of a photochromic ink based on benzylpyridines activated by UV light, which makes them turn a dark blue color. Then, benzylpyridines become progressively lighter over time and even if the ambient temperature rises
CoolVu Food^®^ (www.product.statnano.com)	Dairy products and beverage	Over a period of time, an active zone fades from silver to white. The higher the storage temperature, the faster the fading
Smart dot (www.evigence.com)	Bakery and frozen products	Indicator changes color from green to red when exposed to temperature
WarmMark^®^ (www.deltatrak.com)	Shipping, storage, processing	Visual pass/fail confirmation of exposure to temperature excursions. It is a blotter paper pad saturated with a red-dyed chemical
Cold Chain iToken^TM^ (www.deltatrak.com)	Supply chain	Simple pull-tab activation provides positive “ON” reading; can be scanned with barcode readers
TempDot^®^ (www.deltatrak.com)	Seafood and meat	Indicator window confirms activation; labels can be shipped and stored under any temperature
Freshtag/Check point ^®^ (www.vitsab.com)	Meat, fish and dairy products	The indicator has two separate compartments: enzyme solution compartment and substrate solution compartment and pH indicator; controlled lipolytic hydrolysis of substrate by enzymes triggers pH reduction and color change from green to red
OliTec^TM^ (www.oli-tec.com)	Fresh products	It is a multi-layer label that can monitor degradation profiles of food products at specific storage conditions
TOPCRYO (www.cryolog.com)	Cold chain	It can monitor cold chain compliance; microbiological label changes its color from green to red
Traceo^®^ (www.cryolog.com)	Chilled food products	It is based on a microbiological system. It is a transparent adhesive label in which selected strains of lactic acid bacteria are trapped. It delivers a clear twofold response: an irreversible change from colorless to pink and a simultaneous opacification reaction once the product has experienced critical temperature abuses or once it has reached its use by date
eO^®^ (www.cryolog.com)	Cold chain	It is based on a microbiological system. It is an adhesive label in the form of a small gel pad shaped like the petals of a flower that change from green to red color. The color change represents a pH change due to microbial growth of lactic acid bacteria
Keep-it^®^ (www.keep-it.com)	Fresh products, especially fish	The content of the indicator is specifically tailored to different products, and simulate how the product’s remaining shelf-life is reduced over time. When the product is stored where it is warm, the indicator will move rapidly. When the product is kept cold, the indicator will move slowly. When the indicator shows zero, the product is no longer edible
FreshCode^®^ (www.freshcodelabel.com)	Poultry products	The white center of the indicator is impregnated with an intelligent ink, which captures the emission of volatile gases released during spoilage of chicken in modified atmosphere packaging. The product is no longer suitable for consumption when the indicator turns fully black
Tempix^®^ (www.tempix.com)	Cold chain	The black bar in the indicator ensures that the product has been kept at the correct temperature throughout the cold chain. Should the product have been exposed to temperatures above the recommended limit at any stage of its handling, the black bar will disappear from the window

**Table 5:** Commercially available freshness indicators.

Commercial Name	Application	Principle	Way of Acting
Raflatac (www.upmraflatac.com)	Poultry	It is based on a nanolayer of silver that reacts with hydrogen sulfide, a breakdown of cysteine	The indicator is opaque light brown at the moment of packaging, as silver sulfide is formed the color of the layer is converted to transparent
RipeSense^®^ (www.ripesense.co.nz)	Fruit	It detects aroma components or gases involved in the ripening process (e.g., ethylene)	The label is initially red and graduates to orange and finally yellow as the ripening progresses
Food fresh^TM^ (www.vanprob.com)	Meat	It is an irreversible time monitoring self-adhesive label indicators that can be set to time out within a given ‘consume within’ time frame, ranging from a few days to months.	The indicator contains a porous membrane, through which a colored liquid travels at a pre-calibrated rate. It is activated by squeezing the bubble placed above. A red line appears almost immediately to confirm that the indicator has started monitoring

**Table 6:** Commercially available gas indicators.

Commercial Name	Application	Principle	Way of Acting
Ageless Eye^®^ (www.mgc.co.jp)	Meat	These are sachets contain an oxygen indicator tablet	When oxygen is absent in the headspace, the indicator displays a pink color. When oxygen is present, it turns blue
Tell-Tab^TM^ (www.impakcorporation.com)	All products	It is an in-package monitor which indicates the presence of oxygen	When exposed to oxygen, the system turns blue or purple, then returns to its original pink color as the oxygen in the container is reduced
O_2_Sense^TM^ (www.evigence.com)	Fresh products	An eye readable indicator to detect leakages in modified atmosphere packaging MAP.	It acts by means of a color change
Novas Insignia Technologies (www.insigniatechnologies.com)	Products packed in a modified atmosphere	It shows when packaging has been damaged. This allows manufacturers and retailers to remove this product from the supply chain	A specialized pigment for use in plastic packaging shows a clear color change
Shelf Life Guard (www.upm.com)	Meat	The consumer is informed if air has replaced the modified atmosphere gases within the package due to a breach or leak	It acts by means of a color change based on the indicator’s red-ox dye reacting with oxygen between the labeling layers from transparent to blue

**Table 7:** Commercially available sensors.

Commercial Name	Application	Principle
O2xyDot^®^ (www.oxysense.com)	All products	Optical sensor placed in transparent or semi-transparent packages to measure oxygen with sensitive, rapid and non-destructive measurements
Flex Alert (www.flex-alert.com)	Coffee beans, dried nuts, seeds, wine barrels and fresh fruit	Flexible biosensor to detect toxins in packaged foods throughout the supply chain. It has been specifically developed against *Escherichia coli*, *Listeria* spp., *Salmonella* spp., and aflatoxins

**Table 8:** Commercially available radio frequency identification systems (RFID) technology.

Commercial Name	Application	Principle
Easy2log^®^ (www.environmental-expert.com)	Seafood, meat and poultry, milk-based products, frozen food	It is a low cost, semi-passive tag that allows monitoring temperature-sensitive products during transportation and storage. The tag is also able to calculate the Mean Kinetic Temperature and user configurable remaining shelf-life time as well as generate alarms in case these parameters exceeded user defined thresholds
CS8304 (www.convergence.com.hk)	Cold chain	The tag provides 10,000 samples of logging memory for saving of temperature data. LED light indicates temperature violations
TempTRIP (www.temptrip.com)	Cold chain	This temperature tag uses ultra-high frequency to communicate wirelessly to readers that send the results directly to Internet Web page
